# The Challenge of Additionality: The Impact of Central Grants for Primary Healthcare on State-Level Spending on Primary Healthcare in India

**DOI:** 10.15171/ijhpm.2019.06

**Published:** 2019-02-18

**Authors:** Diana M. Bowser, Rajesh Jha, Manjiri Bhawalkar, Peter Berman

**Affiliations:** ^1^ The Heller School for Social Policy and Management, Brandeis University, Waltham, MA, USA.; ^2^ Independent Consultant, Delhi, India.; ^3^ Health Financing Team, The Global Fund to Fight AIDS, Tuberculosis and Malaria, Geneva, Switzerland.; ^4^ Department of Global Health and Population, International Health Systems Program, Harvard School of Public Health, Boston, MA, USA.

**Keywords:** Health Systems, Spending and Financing, Primary Healthcare, India, Fungibility

## Abstract

**Background:** In planning for universal health coverage, many countries have been examining their fiscal decentralization policies with the goal of increasing efficiency and equity via "additionalities." The concept of "additionality," when the government of a lower administrative level increases the funding allocated to a particular issue when extra funds are present, is often used in these contexts. Although the definition of "additionality" can be used more broadly, for the purposes of this paper we focus narrowly on the additional allocation of primary healthcare expenditures. This paper explores this idea by examining the impact of central level primary healthcare expenditure, on individual state level contributions to primary healthcare expenditure within 16 Indian states between 2005 and 2013.

**Methods:** In examining 5 main variables, we compared differences between government expenditures, contributions, and revenues for Empowered Action Group (EAG) states, and non-EAG states. EAG states are normally larger states that have weaker public health infrastructure and hence qualify for additional funding. Finally, using a model that captured the quantity of central level primary healthcare expenditure distributions to these states, we measured its impact on each state’s own contributions to primary healthcare spending.

**Results:** Our results show that, at the state level, growth in per capita central level primary healthcare expenditure has increased by 110% from 2005-2013, while state’s own contributions to primary healthcare expenditure per capita increased by 32%. Further analyses show that a 1% change disbursement from the central level leads to a -0.132%, although not significant, change by states in their own expenditure. The effect for wealthier states is -0.151% and significant and for poorer states the effect is smaller at -0.096% and not significant.

**Conclusion:** This analysis suggests that increases in central level primary healthcare expenditure to states have an inverse relationship with primary healthcare expenditures by the state level. Furthermore, this effect is more pronounced in wealthier Indian states. This finding has policy implications on India’s decision to increase block grants to states in place of targeted program expenditures

## Background

As fiscal policies evolve and countries around the globe plan for universal health coverage, countries are re-examining their fiscal decentralization policies.^1-3^ In many contexts, some form of fiscal federalism will be contemplated as part of the reform process. Fiscal federalism is defined as both the conditional or unconditional allocation of funds by a central or federal government to lower level provinces or states and often involves a level of political and administrative decentralization with regard to specific programs, such as healthcare.^4^ In countries such as Nigeria, Ethiopia, South Africa, and India, subnational levels are already responsible for a significant portion of the total health spending in the country. For example, in India, as of 2007, 68% of public health spending is executed at the state or lower level.^5^

Research and analyses surrounding fiscal federalism and incentives, has examined many outcomes, including changes in both redistribution and efficiency.^2,5,6^ Much of the literature on fiscal federalism and other forms of decentralization has focused on how allocations have improved inequalities across lower-level entities.^7-10^ Another rich source of literature examined the important policy question of whether lower levels maintain or ement spending in a particular area as a result of receiving additional funds, sometimes referred to as “additionality”or “crowding in.”^11,12^ Research on the additionality effect at the national level has shown that countries reduce their domestic funding for health in response to increased donor funding for health.^11,13,14^ Similar research on the crowding effect, also finds varying patterns of lower level expenditures given changing federal allocations.^11^ Given these are similar ideas, for the purpose of this paper we will refer to the use of additional funds as “additionality.” All of the research above has focused on an attempt to answer what the patterns or deviations from the norms are, given changing relationships between the central and lower levels. This paper uses variations in central level expenditure across different states in India to understand the relationship between central expenditure and lower-level spending on primary healthcare.

Understanding the effects of fiscal federalism on overall resource allocation is important in its own right. However, the concept is even more important as it relates to subnational spending on primary healthcare. The Lancet’s “Global Health 2035” Commission has stressed the importance of investments in primary healthcare and public health systems for improved population health, health equity, and economic development in low- and middle-income countries.^15^ Although the Commission more generally highlights the improvements that follow from investments in health, the Commission specifically emphasizes the substantial benefits that arise from investing in primary healthcare.^15^ In addition, a focus on primary healthcare is essential for achieving universal health coverage.^16-19^ Karan et al have found that the Indian government, despite a health insurance package targeted at the urban poor, has failed to protect low-income populations from catastrophic health spending.^18^ Out of pocket expenditures as a percentage of central health expenditure in India is as high as 65% in 2015, and has remained at that level since 2010.^20^ These results provide an important justification for increasing spending in countries like India, where minimal financial investments in health can not only yield large economic benefits, but also increase access, and reduce out-of-pocket payments.^15^

Primary healthcare is mainly the responsibility of states and lower level entities in India.^21^ Within India’s government health system, responsibilities for health and health-related services are largely with state health departments and their subordinate units. There are important functions related to nutrition or sanitation and water supply that are more focused in other departments. Federal financing makes up about one third of overall government health financing with states contributing most of the rest with incorporation of some central support as discussed in this paper. Recent years have seen efforts to increase the role of local government institutions (Panchayati Raj Insitutions or PRIs), especially in community public health functions.^22^ PRIs do receive some support from government departments including health (under National Health Mission [NHM]) and others, however financing for PRIs do not account for a large share of government health financing. Understanding how central level transfers for primary healthcare affect state level expenditure on primary healthcare is key to developing more effective national policies.

There are a number of studies that examine patterns of fiscal federalism as it relates to healthcare spending in countries around the globe.^5,6,23^ Several of these papers have focused on India, as it is one of the largest federal states where a significant percentage of central healthcare funding is allocated to the lower level.^5^ One study examines fiscal federalism and the subsequent substitution effect in India, showing a negative relationship between central allocations and state level expenditure. This is a critically important study on this topic in India, and provides sound analytical econometric modeling that can be updated and enhanced with the most recent data.^24^ In using the most recent data available, this paper adds to the literature by empirically examining both the impact of fiscal federalism on Indian state and subnational level expenditure in a large country using data across a large number of years.

### Fiscal Federalism in India

Government healthcare in India is principally financed by individual states. However, given the large variability in state governments’ spending abilities, the central government allocates substantial transfers of funding for health to the states. Most of the central government’s transfers are for primary healthcare and come through the NHM (originally the National Rural Health Mission, NRHM), which focuses on providing healthcare to vulnerable populations and to states with greater financial need.^25^ Thus, central transfers are important for financing primary healthcare, both in terms of their direct contributions but also in terms of how they affect resource allocation decisions to primary health in the individual states.

The initial NRHM, launched on April 12, 2005 by the Government of India, was focused on 18 Empowered Action Group (EAG) states, which are Indian States that have been identified as larger, poorer states with weak public health indicators and infrastructure that qualify for additional funding and programs (Bihar, Chattisgarh, Jharkhand, Madhya Pradesh, Orissa, Rajasthan, Uttarakhand, Uttar Pradesh, Arunachal Pradesh, Assam, Manipur, Mizoram, Meghalaya, Nagaland, Sikkim, Tripura, Jammu and Kashmir, Himachal Pradesh). EAG and non-EAG States differ with regard to a number of health metrics. For example, life expectancy in non-EAG states is around 72 years, while in EAG states it is about 68 years.^26^ Similarly, the maternal mortality ratio is around 85 maternal deaths per 100 000 live births in non-EAG states and it is 188 maternal deaths per 100 000 live births in EAG states.^27^ The focus of the NRHM was to expand access to priority primary healthcare services through increased central grants to states.^28^ Furthermore, it was also designed to improve efficiency as well as reduce inequalities across states. The NHM included an urban component.

To improve public spending efficiency, there were several different grants from the center dispersed to states. Some grants were tied directly to federally prescribed program designs and others, like NHM, allowed flexible spending designed to address state-specific conditions and priorities. Furthermore, to reduce spending disparities across states, central grants were designed to be more generous to EAG and northeastern states in comparison to others.

To incentivize states to spend more of their own resources on primary healthcare, central grants for NHM were tied to explicit co-funding requirements. However, the amount of the central government transfer to the state level for this initiative has changed over time. It is also different for EAG versus non-EAG states. Between the years 2005-2007, which overlapped with the 10th Five Year Plan (FYP, 2002-2007), the central government contributed 100% of the program funding to all states. Subsequently, under the 11th FYP (2007-2012), the central government contributed 85%, while states were required to contribute 15%. In the current 12th FYP (2012-17), this ratio changed again to 75% central government contribution and 25% state contribution. Exceptions to this rule were introduced for northeastern states and 3 hill states where the ratio was altered to reflect a 90%-10% split.^25^ Following recommendations from the 14th Finance Commission, in the financial year 2015-2016, the financing pattern from the central government reduced from 75% to 60% (40% for states), while the exception for northeastern and hill states continued.

## Methods

### Data

Described below are 5 key variables containing annual data over an 8 year time periods (2005-2013) for 16 Indian state. State’s own contribution to primary healthcare, the main outcome variable of interest, was defined as total expenditure incurred by the state government on primary healthcare out of its own allocations through the Department of Health and Family Welfare (excluding central government allocations). State’s own contribution included both their own revenue from taxes that was used for primary healthcare as well as the central fiscal transfer to the state treasury for primary healthcare. These 2 component could not be analyzed separately for methodological reasons. While there were multiple global definitions of primary healthcare financing, the definition of state level primary healthcare financing from the Department of Health and Family Welfare was used to maintain consistency with Government of India definitions and classifications. Central primary healthcare expenditure was defined as total expenditure at the State level sourced from the central government and given to each State through the Department of Health and Family Welfare for primary healthcare as part of the NHM. Government primary healthcare expenditure (GPHCE) at the state level was defined as total government expenditure on primary healthcare services including state government contributions and central government expenditure through the Department of Health and Family Welfare at the state level. Total government health expenditure at the state level was defined as total government expenditure on health through the Department of Health and Family Welfare from all sources for each state. State’s own revenue was the total tax revenue generated by state governments.

All monetary values were standardized to 2005 constant rupees using the gross state domestic product deflator from the Ministry of Statistics and Program Implementation in India and then converted to USD using the exchange rate conversions extracted for relevant years from the World Bank conversion database.^29^ Per capita figures were created by dividing total values by the appropriate total population for each India state according to the National Population Commission. All data were examined for EAG and non-EAG states to understand whether spending by lower levels on health was increased or decreased by varying transfers from the central level. The 8 EAG states included in the analysis, defined as larger, poorer states with weak public health indicators and infrastructure that qualified for additional funding and programs, considered in our analysis were Assam, Bihar, Chattisgarh, Jharkhand, Madhya Pradesh, Odisha (formerly Orrisa), Rajasthan, and Uttar Pradesh. The non-EAG states were Andhra Pradesh, Gujarat, Karnataka, Kerala, Maharashtra, Punjab, Tamil Nadu, and West Bengal. Sixteen states were included in the analysis using a convenience sampling technique that allowed for variation by population size and urban and rural states. The sample was also selected based on data availability on healthcare spending for primary healthcare.

All data were extracted from official Government of India databases, including the Reserve Bank of India as well as Official Financial Reports from the NHM and Ministry of Health. The definition and methodology for allocating spending to primary healath care and non-primary healthcare were taken directly from the National Health System Resource Center using the Budget Tracking Toolkit for additional details on budget classification.^30^

### Methodology

The data were first examined qualitatively through several exhibits. The first exhibit examined trends every other year over the period 2005-2013, in 2005 constant USD, for the 5 main variables of interest (state’s own contribution to primary healthcare, central primary healthcare expenditure, GPHCE at the state level, total government health expenditure at the state level, and state’s own revenue) as well as gross state domestic product, ratio of GPHCE at state level divided by total government health expenditure at state level, and population size for the 16 states included in the analysis. The second exhibit examined the per capita form of state’s own contribution to primary healthcare, central primary healthcare expenditure, GPHCE at the state level, and total government health expenditure at the state level for EAG and non-EAG states. The third exhibit examined the correlation between yearly changes in central primary healthcare expenditure per capita and state’s own contribution to primary healthcare per capita for EAG and non-EAG states. As part of the qualitative analysis, trends in state’s own contribution to primary healthcare per capita were quantified for those EAG and non-EAG states with lower and higher yearly changes in central primary healthcare expenditure per capita.

Finally, we used a model that captured the quantity of central primary healthcare expenditure distributions to the states in USD in state *i* at time *t*, and measured its impact on state’s own contribution to spending on primary healthcare from its own funds in USD in state *i* at time *t*, according to:

*Stateown*_it_ *= α + β*_1_ *central*_it_ *+ β*_2_ *GSDP*_it_ *+ β*_3_ *LagStateown*_it-1_ *+ β*_4_ *rev*_it_ *+* f_i_* + d*_t_* + ε*_it_

Where ‘*Stateown*_it_’ was the state’s own contribution to primary healthcare per capita, ‘*central*_it_’ was the central primary healthcare expenditure per capita, ‘*GSDP*_it_’ represented the gross state domestic product per capita in each state at time *t*, ‘*LagStateown*_it_’ was the one year lag of state’s own contribution to primary healthcare per capita, ‘*rev*_it_’ represented each state’s own tax revenue generated by state governments per capita net the state’s own contribution to primary healthcare, ‘f_i_’ were state fixed effects, ‘*d*_t_’ were nationwide time dummies, and ‘*ε*_it_’ was an error term. As the relationship was not perfectly linear, all variables were log transformed for the fixed effects estimation.

Fixed effects were used as the statistical method in order to control for time-invariant unobservable characteristics of each state and exploit within group variation across time in order to estimate the coefficient of interest. The benefits of using this model were that characteristics of states that may influence the relationship between central primary healthcare expenditure and states’ own contribution that were also difficult to measure could be proxied through a fixed effects estimator for each state, thereby eliminating variation across states, which was a potential source of omitted variable bias. A limitation was that if the model did not capture all factors that influence state spending on health at the state level, then it could worsen remaining bias. The time dummies ‘*d*_t_’ accounted for changes in state’s own contribution to primary healthcare per capita over time.

The model measured the degree to which lower administrative levels maintain or supplement spending in a particular area (“additionality”) as a result of receiving additional funds in said area. We examined the response of state level primary healthcare expenditure as a result of additional central level expenditure on primary healthcare to the state level.

We estimated this equation over 8, one-year intervals (2005-2013) within those 16 Indian states for which we had measures of both central and state-level primary healthcare expenditure. We used a fixed-effects panel data model to estimate the impact of central allocations to public health on each state’s subsequent own allocations to public health. The error term ‘*ε*_it_’ may be correlated over time and we therefore clustered the standard errors at the state level in our reported results. The choice between a fixed and random-effect model was informed by a Hausman specification test, which suggested that individual fixed effects were correlated with other predictor variables. Variations of the main models were included in Supplementary file 1 (levels, interactions, and generalized method of moments).

## Results

Table 1 presents the mean and standard deviation across the 16 states included in the analysis for the main variables included in the model using real values (2004-2005) as well as population size. State’s own contribution to primary healthcare expenditure per capita increases by 32%, from 1.53 USD to 2.01 USD per capita over the period 2005-2013 in real terms. Central primary healthcare expenditure per capita at the state level increased by 110% from 0.65 USD to 1.35 USD per capita over the period 2005-2013 in real terms. State’s own contribution to primary healthcare as a percent of the GPHCE varies between 56% and 68% over the time period. Central primary healthcare as a percent of the GPHCE varies between 32% and 44% over the time period. Table 1 tracks the changes in each indicator relevant to the analysis.

**Table 1:** Mean (Standard Deviation) of Model Variables and Population Size^a^

**Variable**	**2005**	**2007**	**2009**	**2011**	**2013**
GPHCE per capita at the state level (2005 USD)	2.18 (0.53)	2.97 (0.92)	3.47 (0.93)	3.98 (1.15)	3.37 (1.02)
Central primary healthcare expenditure per capita (2005 USD)	0.65 (0.17)	1.12 (0.74)	1.52 (0.61)	1.56 (0.64)	1.37 (0.51)
State’s own contribution to primary healthcare expenditure per capita (2005 USD)	1.53 (0.48)	1.84 (0.55)	1.95 (0.56)	2.43 (0.73)	2.01 (0.63)
Central primary healthcare as a % of government primary healthcare at the state level	32% (11%)	37% (14%)	44% (10%)	39% (9%)	41% (7%)
State’s own contribution to primary healthcare as a % of government primary healthcare at the state level	68% (11%)	63% (14%)	56% (10%)	61% (9%)	59% (7%)
State’s own tax revenue per capita (2005 USD)	45.09 (23.43)	54.70 (28.67)	50.53 (25.33)	65.95 (35.20)	61.80 (32.45)
Population	63 356 250 (39 820 179)	65 205 313 (41 314 683)	67 015 500 (42 848 511)	68 786 250 (44 380 155)	70 517 750 (45 905 117)

Abbreviation: GPHCE, Government primary healthcare expenditure. ^a^Sample size for each variable for each year is N=16 except for year 2007 where Andhra Pradesh had missing figures for the following indicators: GPHCE at the state level, GPHCE per capita at the state level, state’s own contribution to primary healthcare expenditure and state’s own contribution to primary healthcare expenditure per capita; and year 2007 where Jharkand and Kartanaka had missing figures for the following indicators: state’s own tax revenue and state’s own tax revenue per capita missing for Jharkand in 2007 and Karnataka in 2009. Exchange rate conversions were extracted for relevant years from the World Bank conversion database.^29^

**Figure 1:**
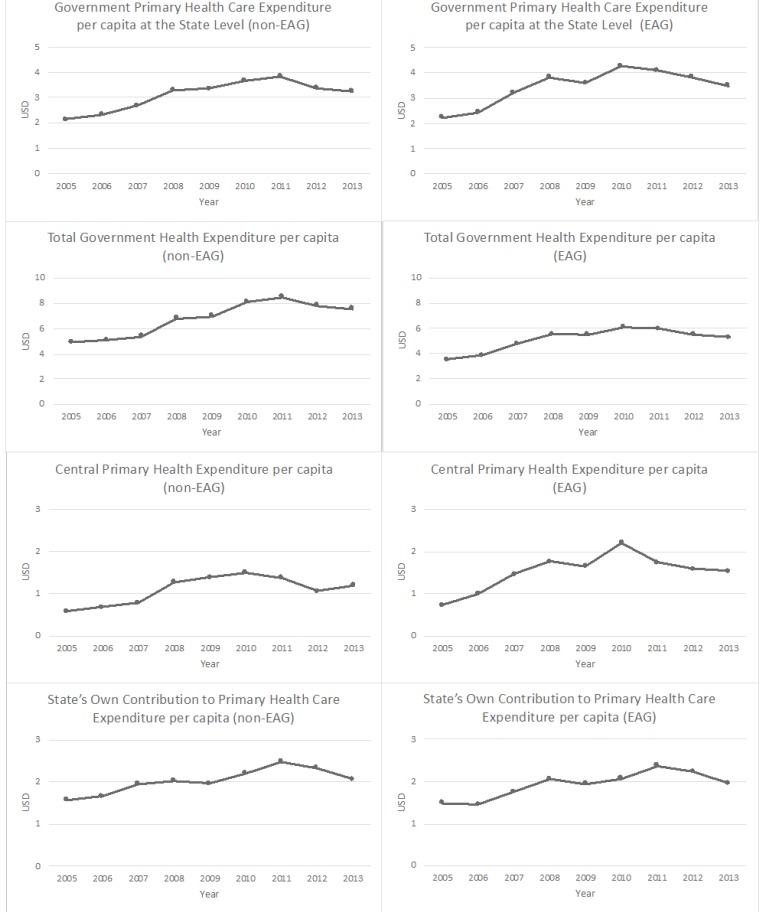
Average Per Capita Central and State Level Government Expenditures for EAG and non-EAG Indian States, 2005-2013, (2005 USD). Abbreviation: EAG, Empowered Action Group.

**Figure 2:**
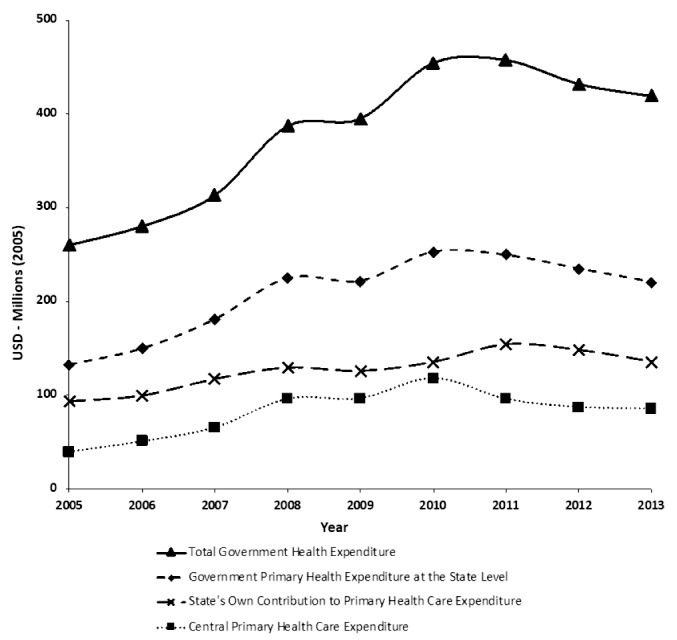
Average Total, Central and State Level Government Expenditures for 16 Indian States, 2005-2013, Million (2005 USD).

Figure 1 shows the expenditure per capita averaged across all 16 states, divided into EAG and non-EAG groups. For all states, both total government health expenditures and GPHCE at the state level are trending upwards over the period 2005 to 2013. The growth is even more rapid among non-EAG states. The figure shows that while central primary healthcare expenditure is trending upwards over the entire time period, there is a reduction in central allocations in 2011 and 2012, as a results of national policies on allocation that are examined empirically below. State’s own contribution to primary health expenditure rises steadily over the entire time period from 2005 until 2013. Spending levels remain fairly flat in the early years, which is most likely due to a slowing in allocations from the center due low initial budget execution for some states. We also repeat this analysis looking at real expenditures averaged across all states, which follows a similar pattern. This graph can be seen in Figure 2.

### Modeling Results

Table 2 quantifies how the magnitude of the expenditure on primary healthcare at the state level is affected by the magnitude of the transfers from the central level. The model relates the quantity of central primary healthcare expenditure distributions to the states in USD over the time period 2005-2013, and measures the impact on state’s own contribution to primary healthcare expenditure from its own funds in USD. As shown in Table 2, a 1% increase in central primary healthcare expenditure per capita is associated with a 0.151% reduction in state’s own contribution to primary healthcare expenditure per capita (*P* < .01), controlling for log of per capita GSDP, lagged levels of state’s own contribution to primary healthcare, log of state’s own tax revenue per capita net primary healthcare revenues, as well as state fixed effects and time trends. For all states combined and non-EAG states, the magnitude and direction of change are the same as non-EAG states, but the results are not significant. How much each state prioritizes primary healthcare expenditure, proxied with lagged levels of state’s own spending on primary healthcare, has a significant positive impact on state’s own contribution to primary healthcare expenditure per capita, which is significant for all states and non-EAG states. This may reflect some type of expenditure momentum, in that most states’ own contribution to primary healthcare expenditure per capita is for non-discretionary items like salaries.

**Table 2:** Results of Model Relating Central Primary Healthcare Expenditure Per Capita to State’s Own Contribution to Primary Healthcare Expenditure Per Capita, 2005 USD, 2005-2013

	**Log of State’s Own Primary Healthcare Expenditure Per Capita, (2005 USD)**	**Log of State’s Own Primary Healthcare Expenditure Per Capita, (2005 USD) Non-EAG**	**Log of State’s Own Primary Healthcare Expenditure Per Capita, (2005 USD) EAG**
Log central primary healthcare expenditure per capita	-0.132 (0.084)	-0.151 (0.041)***	-0.096 (0.179)
Log GSDP per capita	0.449 (0.478)	1.552 (0.618)**	-0.016 (0.802)
Log state’s own primary healthcare expenditure per capita lagged (t-1)	0.255 (0.131)*	0.405 (0.081)***	0.111 (0.168)
State’s own tax revenue per capita	-0.608 (0.481)	-0.549 (0.344)	-0.786 (0.592)
Constant	-0.006 (3.115)	-7.871 (3.835)*	3.425 (4.523)
N	125	61	64
States	16	8	8
R2	0.51	0.76	0.44

Abbreviations: GSDP, gross state domestic product; EAG, Empowered Action Group. * *P *< .1; ** *P *< .05; *** *P *< .01, cluster robust standard errors in parentheses.

## Discussion

The results above show important new evidence of how India’s central GPHCEs passed on to Indian states, impact states’ own contributions to primary healthcare expenditure. We find evidence of a lack of additionality of central funding across all Indian states. On average during this period, states reduce their own spending by 0.132% for every 1% increase in central funding, although this result is not significant. There is a statistically significant reduction in state’s own spending on primary healthcare, -0.151%, for non-EAG Indian States, and statistically not significant for EAG states. Overall, we find no evidence that receiving additional funds from the central level stimulates stable or increasing funding by states during a period of significantly increased central funding.

There are only a few other studies that have examined this relationship between central and state level expenditure on primary healthcare in India. For example, Rao and Choudhury examined the relationship between central primary healthcare expenditure and each state’s own contributions to primary healthcare expenditure between 1991 and 2007 in 14 Indian states.^1^ They also found a significant negative effect in the range of -0.91 for the period 1991 to 2007 and -1.059 for the period 2001-2007. The coefficient found in this paper is smaller, most likely due to using a log transformed model.^24^ When the above model is not transformed and only more wealthy states are included, the coefficient approaches that of (-0.41).^24^ Comparing our results to these older results are interesting for 2 reasons. First, there seems to be a change in additionality for poorer states, as their coefficient is not statistically different from zero in our current analysis, and may have been more negative in the past. Secondly, the overall lack of additionality may be reducing over time, as states may be increasingly likely to allocate more resources to primary healthcare in addition to central level expenditure.

Hooda’s analysis examines general public health expenditures in India, finding that some states are not able to absorb and use all the funds allocated to them by the central government in a timely way.^31^ Therefore, these states may differentially have to spend their own, additional revenues to support primary healthcare. Those states that are better able to absorb and use central funds can better allocate their own funds away from health. Hooda suggests that those states that are not able to spend allocated funds are hindered by issues of inadequate human resources, poor infrastructure and weak technical capability.^31^

Related work examining individual states’ spending has shown that wealthier states are better able to use central allocations and over time have captured more of them.^30^ Wealthier states’ ability to capture more central expenditure for primary healthcare is likely to be associated with shifting more of their own spending to non-primary healthcare uses (ie, less additionality to primary healthcare). It is possible that states are responding to changes in central grants by treating tied funds more like general revenue through displacing their own funding away from primary healthcare.

Berman, Bhawalker, and Jha qualitatively examine some of the mechanisms for additionality in poorer and richer states, as quantitatively modeled above. They report that although poorer states were given greater opportunities to use increased central funds, they generally were less able to do so, often due to failings in public financial management.^30^ This undermines the mission of targeted support for primary healthcare in the lagging states and results in increasing spending differentials across states, even though a key goal of central spending is to reduce such disparities.

Our results suggest that central funds overall were not successful in incentivizing increases in state spending. In wealthier states, lower additionality was found as states diverted expenditures away from primary healthcare as central expenditures increased. In poorer states, evidence for this substitution was not conclusive. Overall, the fiscal mechanisms of the NRHM/NHM do not seem to have consistently achieved their objectives. This is in part because in EAG states have a higher burden and need for primary care services, where as in non-EAG states, who often have a more robust tertiary care system, they may face pressures to divert expenditure away from primary healthcare to other health needs.

Given the central government’s stated commitment to equity and pro-poor policies, they may need to reconsider methods used to allocate funds to states for primary healthcare. The factors that influence states’ behavior, as partially examined by Hooda and Berman, also need to be examined in more detail.

One approach to assuring additionality has been to require states to increase their own spending in proportion to central grants, as in the current form of fiscal decentralization in India. However, this may be difficult to monitor and enforce without explicit accounting standards to measure before and after spending. Frequent elections and changes in political alignment in India also make enforcement less reliable.

An alternative approach is to make future central allocations proportional to, and conditional on, increased state allocations. The central government could propose offsetting a percentage of increased state budget plans. However, this would place the poorer states at a disadvantage given their more limited fiscal capacity. One approach reflected in recent years has been to provide more central funding in the form of untied block grants to states, giving them a greater role in overall resource allocation across sectors (health vs. non-health) as well as within sectors (primary healthcare vs. hospitals, for example). It is unclear what effect this might have on states’ primary healthcare spending overall.

Our results control for several important factors including fixed effects as well as GSDP, the ratio of GPHCE at state level/total government health expenditure at state level, and state’s own tax revenue per capita. Controlling for fixed effects is important to show that even in states with similar levels of some of the unmeasurable factors that might impact primary healthcare expenditure, such as motivation, states reduce their own expenditure to primary healthcare depending on central level expenditure. Our results reflect including GSDP in the model, suggesting that GSDP differences alone are not enough to explain the reduction in states’ own investments in primary healthcare.

Our results are subject to limitations of data availability and data quality. The requisite data on state expenditures and central government transfers are not available for all states, thus our estimates are only valid to the states included in our sample and may not be generalizable to other states. By introducing a differenced two-way fixed effects model, we are able to control for all state-level effects that are constant by year and all year-level effects that are constant by state, which eliminates the burden of some omitted variables. However, as a result of these modeling choices, the estimated relationships should not be applied to any single state in any single year but rather be taken as a look at homogenous effects across states and years. For this same reason, there may be some selection bias in the models as those states with better and available data are the ones included in the analysis. In addition, the analysis focuses exclusively on spending for primary healthcare and did not include spending on higher-level services financed through schemes such as the Rashtriya Swasthya Bima Yojana. Finally, our analysis should not be taken as the demonstration of a causal effect between predictors and outcome. However, we do believe that our findings are in line with basic economic theories as well as past research on additionality in total health expenditure and the fungibility of other types of aid. Our key findings are a significant step in exploring the fungibility of central grants to primary healthcare using the best data available at this time.

## Conclusion

This paper evaluates the degree of additionality provided by central grants for primary healthcare in India. Our analysis suggests that there is a lack of an additionality effect between central allocations and state’s own contribution to public health, especially in wealthier states. The effect in wealthier states is negative and greater than random variation would predict. While still negative in poorer, more populous states, we cannot distinguish this from no effect at all. Given the importance of increasing investment in primary healthcare to address health equity and population health outcomes, these results suggest that central government efforts to affect state spending and priorities need further development. In addition, the relationship between additionality and the ability to absorb and spend central level funds is an important future research question. This finding is especially relevant today when the central government has significantly increased block grants to the states in place of targeted program expenditures.^5^

## Acknowledgements

This work was supported through the Resource Tracking and Management Project to Harvard T. H. Chan SPH from the Bill and Melinda Gates Foundation - Grant #OPP1081405. The authors would like to thank David Kapaon for his assistance in the preparation of this manuscript.

## Ethical issues

Ethical approval was not needed as all data used in the analysis were publicly available.

## Competing interests

Authors declare that they have no competing interests.

## Authors’ contributions

Literature review and background data collection was completed by DMB, MB, and RJ. Data extraction was completed by MB and RJ. DMB and PB analyzed and interpreted the data. DMB wrote the manuscript. Manuscript review and editing was completed by DMB, PB, MB, and RJ. DMB, PB, MB, and RJ all worked on the final version to be published.

## Authors’ affiliations

^1^The Heller School for Social Policy and Management, Brandeis University, Waltham, MA, USA. ^2^Independent Consultant, Delhi, India. ^3^Health Financing Team, The Global Fund to Fight AIDS, Tuberculosis and Malaria, Geneva, Switzerland. ^4^Department of Global Health and Population, International Health Systems Program, Harvard School of Public Health, Boston, MA, USA.

## Supplementary files

Supplementary file 1 contains Tables S1-S3.

## Key messages

**Implications for policy makers**

- Block grants allocated to lower levels for healthcare services, such as the National Health Mission (NHM) in India, can influence lower level spending on health.
- Examining the relationship between cental allocations and lower level spending is important to understand the impact of these financing mechanisms.
- More specifically, findings from this study are important to Indian policymakers in assessing how funds disbursed at the central level can be better allocated to lower levels in order to both, achieve universal health coverage, and improve efficiency, and equity for poorer states in the coming years.

**Implications for public**

Findings from our study demonstrate that in India, “additionalities” for primary healthcare persist when extra funds are present. Though focused solely on India, this study has policy implications, especially for other federal states and those countries that are in the process or considering decentralization. These results reveal a disconnect between the spending priorities at different levels of government, demonstrating that decisions on whether to maintain or supplement funding for specific programs depend on varying factors across Indian states. Given that the general public is often the direct recipients of these funds, they both stand the most to gain if targeted areas are supplemented with additional funds, and the most to lose if the funding for such programs is at most maintained, or worse decreased.
