# Supplementary material for: The Challenge of Additionality: The Impact of Central Grants for Primary Healthcare on State-Level Spending on Primary Healthcare in India
Source: Int J Health Policy Manag. 2019 Feb 18;8(6):329–36. doi: 10.15171/ijhpm.2019.06 (PMC6600018; doi:10.15171/ijhpm.2019.06)
Supplement: Supplementary file 1 — contains Tables S1-S3. [file ijhpm-8-329-s001.pdf]

Supplementary file 1

Table S1. Fixed effects estimate of Central Primary Healthcare Expenditure Per Capita on State's Own Contribution to Primary Healthcare Expenditure Per Capita, 2005 USD, 2005-2013

	**State's Own Contribution To Primary Healthcare Expenditure Per Capita, (2005 USD)**
Central Primary Health Care Expenditure per capita	-0.025 (0.142)
GSDP per capita	0.001 (0.001)
Lag State's own contribution to PHC per capita	0.350 (0.180)*
State's Own Tax Revenue per capita	-0.004 (0.005)
Constant	1.047 (7.21)
N	125
States	16
R2	0.52

* *P* < .1; ** *P* < .05; *** *P* < .01, cluster robust standard errors in parentheses.

Table S2. Fixed Effects Estimate of Central Primary Healthcare Expenditure Per Capita on State's Own Contribution to Primary Healthcare Expenditure Per Capita, 2005 USD, 2005-2013

	**State's own contribution to primary health care expenditure per capita, (2005 USD), EAG States**	**State's own contribution to primary health care expenditure per capita, (2005 USD) Non-EAG States**
Central primary health care expenditure per capita	0.068 (0.140)	-0.415 (0.108)***
GSDP per capita	0.002 (0.002)	0.002 (0.001)*
Lag State's own contribution to PHC per capita	0.311 (0.237)	0.358 (0.155)*

State's own tax revenue per capita	-0.020 (0.021)	-0.001 (0.006)
Constant	1.305 (0.909)	0.026 (0.310)
N	64	70
States	8	8
R2	0.46	0.61

* *P* < .1; ** *P* < .05; *** *P* < .01, cluster robust standard errors in parentheses.

Table S3. Fixed effects and Generalized Method of Moments (GMM) Estimates of log of Central Primary Healthcare Expenditure Per Capita on log of State's Own Contribution to Primary Healthcare Expenditure Per Capita With An Interaction Term to Capture EAG versus non-EAG, 2005 USD, 2005-2013

	**Log State's own contribution to primary health care expenditure per capita, (2005 USD), Fixed Effect**	**Log State's own contribution to primary health care expenditure per capita, (2005 USD) GMM**
Log Central primary health care expenditure per capita	-0.135 (0.045)***	-6.842 (5.456)
EAG vs no-EAG	---	0.784 (0.800)
Interaction (Central primary health care expenditure per capita*EAG)	0.008 (0.212)	6.411 (5.099)
Log GSDP per capita	0.452 (0.447)	1.448 (1.292)
Log Lag State's own contribution to PHC per capita	0.255 (0.132)*	1.085 (0.540)**
Log State's own tax revenue per capita	-0.611 (0.423)	0.241 (0.637)
Constant	-0.016 (3.143)	-10.459 (8.446)
N	125	125
States	16	8
R2	0.51	---

* *P* < .1; ** *P* < .05; *** *P* < .01, cluster robust standard errors in parentheses.
